# Supplementary material for: Evidence supported by Mendelian randomization: impact on inflammatory factors in knee osteoarthritis
Source: Front Med (Lausanne). 2024 May 28;11:1382836. doi: 10.3389/fmed.2024.1382836 (PMC11165061; doi:10.3389/fmed.2024.1382836)
Supplement: Supplementary file 1 [file Data_Sheet_1.ZIP › data/GCST90274809_5e6/809.forest.pdf]

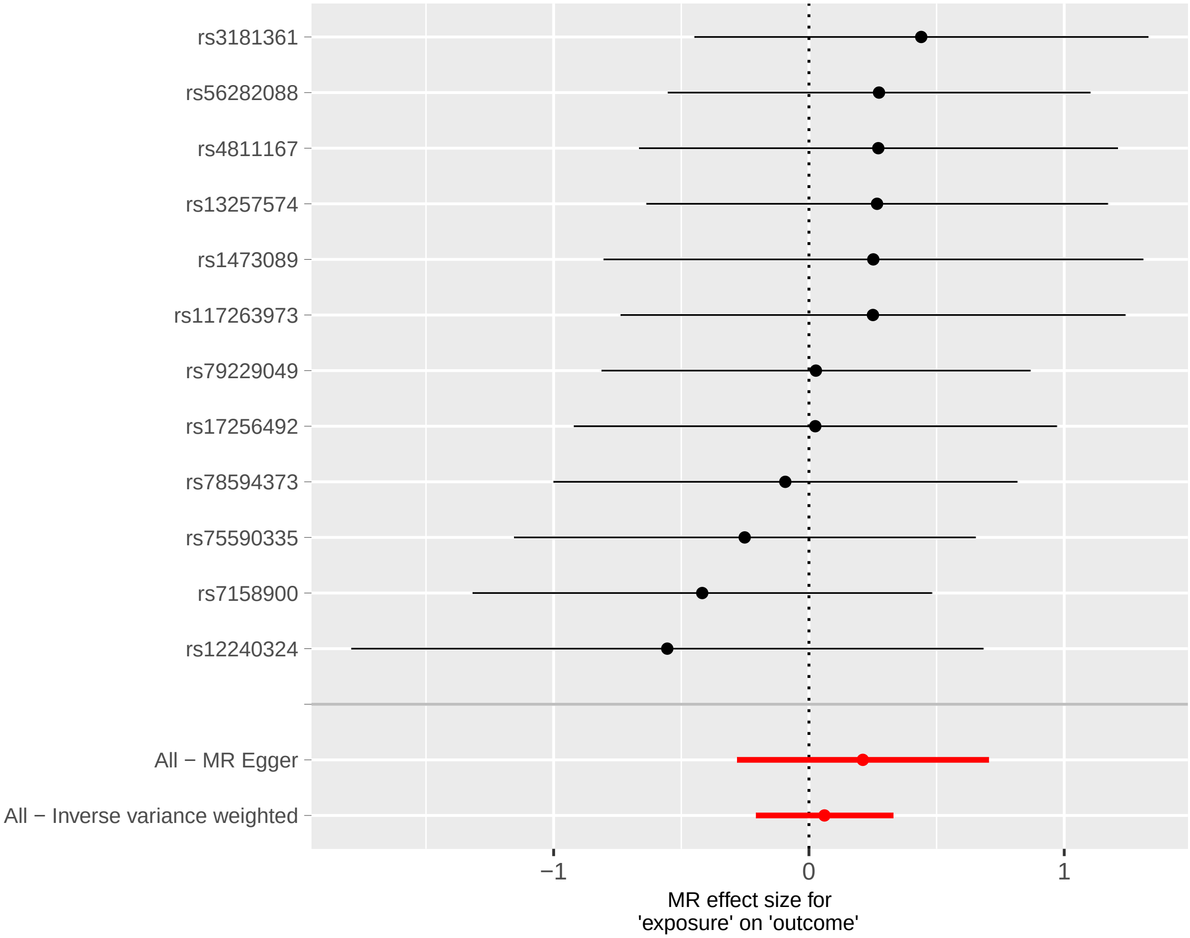

rs3181361
rs56282088
rs4811167
rs13257574
rs1473089
rs117263973
rs79229049
rs17256492
rs78594373
rs75590335
rs7158900
rs12240324
All − MR Egger
All − Inverse variance weighted
−1
0
1
MR effect size for
'exposure' on 'outcome'
